# DNA origami based Au–Ag-core–shell nanoparticle dimers with single-molecule SERS sensitivity†
†Electronic supplementary information (ESI) available: Additional information about materials and methods, designs of DNA origami templates, height profiles, additional SERS spectra, assignment of DNA bands, SEM images, additional AFM images, FDTD simulations, additional reference spectra for Cy3 and detailed description of EF estimation, simulated absorption and scattering spectra. See DOI: 10.1039/c5nr08674d



**DOI:** 10.1039/c5nr08674d

**Published:** 2016-02-19

**Authors:** J. Prinz, C. Heck, L. Ellerik, V. Merk, I. Bald

**Affiliations:** a Institute of Chemistry , University of Potsdam , Karl-Liebknecht-Str. 24-25 , 14469 Potsdam , Germany . Email: bald@uni-potsdam.de; b BAM Federal Institute for Materials Research and Testing , Richard-Willstätter Str. 11 , 12489 Berlin , Germany; c Department of Chemistry + SALSA , Humboldt-Universität zu Berlin , Brook-Taylor-Str. 2 , 12489 Berlin , Germany

## Abstract

DNA origami nanostructures are a versatile tool to arrange metal nanostructures and other chemical entities with nanometer precision. In this way gold nanoparticle dimers with defined distance can be constructed, which can be exploited as novel substrates for surface enhanced Raman scattering (SERS). We have optimized the size, composition and arrangement of Au/Ag nanoparticles to create intense SERS hot spots, with Raman enhancement up to 10^10^, which is sufficient to detect single molecules by Raman scattering. This is demonstrated using single dye molecules (TAMRA and Cy3) placed into the center of the nanoparticle dimers. In conjunction with the DNA origami nanostructures novel SERS substrates are created, which can in the future be applied to the SERS analysis of more complex biomolecular targets, whose position and conformation within the SERS hot spot can be precisely controlled.

## Abstract 2

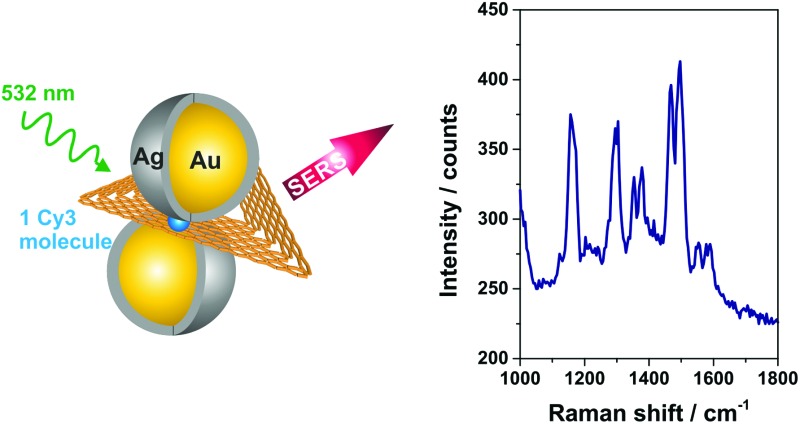
DNA origami nanostructures are used to arrange gold nanoparticles into dimers with defined distance, which can be exploited as novel substrates for surface enhanced Raman scattering (SERS). Single dye molecules (TAMRA and Cy3) can be placed into the SERS hot spots, with Raman enhancement up to 10^10^, which is sufficient to detect single molecules by Raman scattering.

## Introduction

The DNA origami technique introduced by Paul Rothemund in 2006^1^ is a versatile tool that allows for the programmable folding of DNA in various shapes and patterns. Since then, the technique has extensively been applied in order to create for instance three-dimensional structures such as 3D curved structures^2^ or structures with switchable conformations based on DNA regulation.^3^ Recently, a new approach for the formation of higher-order 3D objects *via* shape recognition and without base pairing has been demonstrated.^4^ Since DNA origami templates can be easily modified in numerous ways this technique is frequently used in the field of analytical science. Several DNA origami based studies use atomic force microscopy (AFM) for analytical applications *e.g.* for the detection of inorganic or organic targets,^5^ to analyze enzymatic DNA repair activity^6^ or to study DNA strand breaks.^7,8^ Furthermore, optical methods such as fluorescence spectroscopy are widely used for the analysis of DNA origami templates *e.g.* to perform super-resolution imaging^9^ or to study G-quadruplex folding.^10^

One of the main advantages of DNA origami templates is the possibility of arranging functional units, *e.g.* gold nanoparticles (AuNPs), with nm precision, which makes it a predestined technique for the study of plasmonic effects^11,12^ and surface-enhanced spectroscopies.

Surface-enhanced spectroscopies are based on the enhancement of the electromagnetic field close to metal nanoparticles (NPs) upon excitation of their surface plasmon resonance. This can result in enhanced optical signals such as fluorescence and Raman scattering. Particularly high field enhancement can be generated in the gap between adjacent NPs due to a coupling of the individual surface plasmon resonances.^13,14^ Thus, to benefit from highest field enhancements it is required to control the position of the AuNPs with respect to the analyte molecules.

Depending on the distance to the plasmonic nanostructure a fluorescent dye can be subject to fluorescence quenching^15^ or fluorescence enhancement.^16^ At close distance to the NP surface analyte molecules can also be detected by surface-enhanced Raman scattering (SERS).^17–20^ SERS is a particularly interesting technique since the Raman signal can be enhanced by many orders of magnitude, which renders the detection of single molecules possible.^21,22^

Apart from using ultralow analyte concentrations different approaches aiming at the detection of SERS from single molecules have been developed. On the one hand, the bi-analyte method has been introduced by Le Ru *et al.*,^23^ which is based on the measurement of a mixture of two substances with distinguishable SERS spectra. Subsequently, single-molecule events have to be asserted by a statistical analysis. The bi-analyte method has been improved by isotopic labelling of dyes resulting in spectral shifts of certain SERS peaks.^24^ On the other hand, dimers of Au–Ag-core–shell NPs with tailored gap size have been used for single-molecule SERS. Suh *et al.* presented gap-tailored Au–Ag core–shell nanodumbbells providing enhancement factors (EFs) of the order 10^12^ as well as gold nanobridged nanogap particles generating EF values between 10^8^–10^9^ for about 90% of the enhancing sites.^25,26^ In both cases the gap sizes were in the range of 1 nm and the EFs were high enough for the detection of single Cy3 dyes.

The use of DNA origami structures as scaffolds for SERS active nanostructures is particularly attractive due to their versatility with respect to further functionalization. Additionally, the DNA origami technique represents a bottom-up approach, which allows for the production of a large number of plasmonic nanostructures at once. Such processes are much more cost-effective than widely used top-down lithography methods.^27^ Here, we present DNA–AuNP hybrids that are optimized in various respects in order to increase the SERS sensitivity to a single-molecule level. To the best of our knowledge, this is the first study that combines the DNA origami technique with SERS to detect single molecules. Furthermore, we have estimated EFs for selected nanostructures through direct correlation of AFM and Raman images.

**Fig. 1:**
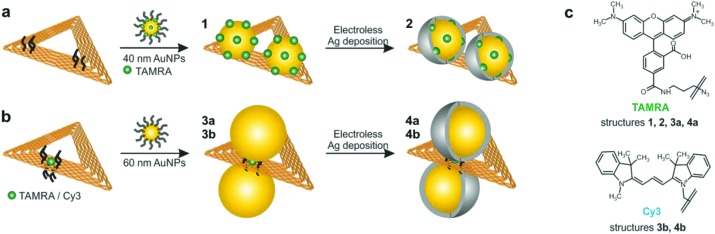
Formation of AuNP dimers using DNA origami substrates. The attachment process is realized by DNA hybridization between ssDNA capture strands protruding from the substrate and the complementary ssDNA coating of the AuNPs. (a) Two 40 nm AuNPs coated with the dye-modified sequence 5′-(TTT)_4_TX-SH-3′ (X = TAMRA) are attached to one side of the DNA origami template resulting in structure **1** with an estimated gap size of 14 nm. Subsequent silver enhancement leads to Au–Ag-core–shell NPs with a decreased gap size (structure **2**). (b) For single-molecule measurements one dye molecule (either TAMRA or Cy3) is incorporated in the DNA origami template. The attachment of two 60 nm AuNPs to different sides of the DNA origami ensures the position of the dye to be exactly in the hot spot (structures **3a**, **3b**). Subsequent addition of a silver layer results in structures **4a** or **4b**. (c) Raman reporter molecules used in the present study with the covalent connection between dye and ssDNA marked in both molecular structures. Structures **1**, **2**, **3a** and **4a** are functionalized with TAMRA whereas structures **3b** and **4b** contain a Cy3 modification.

Here, we present structures based on triangular DNA origami substrates that are functionalized with AuNP dimers. The DNA origami substrates are folded during a hybridization process between the M13mp18 ssDNA scaffold strand and 208 suitable ssDNA staple strands.^1^ By modification of certain staple strands with a capture sequence that protrudes from the DNA triangle anchor points for AuNPs are introduced (see Experimental section for details). Fig. 1 illustrates the attachment process of AuNP dimers to the DNA origami template. Two different strategies (Fig. 1a and b) are pursued that differ in terms of the positions of AuNPs as well as analyte molecules (carboxytetramethylrhodamine (TAMRA), cyanine 3 (Cy3); molecular structures shown in Fig. 1c). In both strategies the attachment process is realized *via* DNA hybridization between the ssDNA capture sequences (5′-(AAA)_8_T_4_-3′) and the ssDNA coating strands (5′-(TTT)_4_T-SH-3′) covering the AuNPs. In strategy (a) two 40 nm AuNPs are attached to one side of the DNA origami template by three anchor points per particle resulting in structure **1**. In that case the AuNPs are covered with a TAMRA-modified sequence (5′-(TTT)_4_TX-SH-3′; X = TAMRA) and a non-TAMRA-modified thiolated DNA strand used as a spacer to reduce the TAMRA concentration on the AuNP surface. In contrast, in strategy (b) one single analyte molecule (TAMRA or Cy3) is incorporated into the DNA origami template by modification of one staple strand. In the following step two 60 nm AuNPs are attached to different sides of the DNA origami template by four anchor points per particle resulting in structure **3a** (TAMRA) or **3b** (Cy3). The different DNA–AuNP hybrids (structures **1**, **3a**, **3b**) are further modified by electroless silver deposition yielding DNA-Au–Ag-core–shell hybrids (structures **2**, **4a**, **4b**). For subsequent Raman and AFM studies the hybrid structures are adsorbed on Si substrates.

**Fig. 2:**
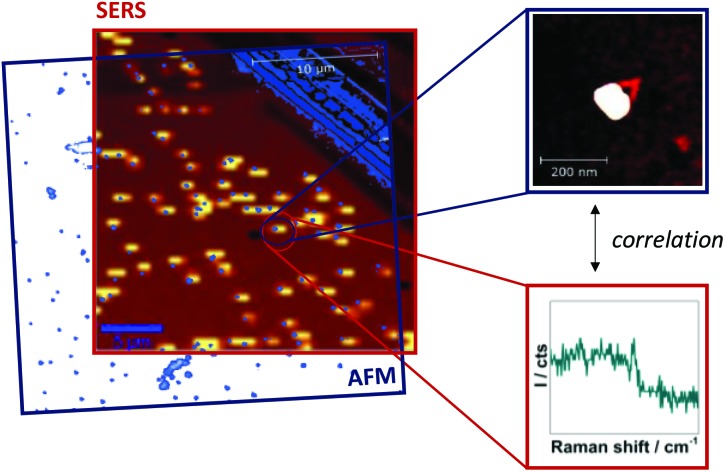
Illustration of correlated AFM and SERS imaging. AFM and SERS images are superimposed using a scratch on the Si as a marker in order to correlate structural and chemical information. In this way SERS signals can clearly be assigned to defined structures (the laser spot diameter is approximately 1.3 μm). For better visualization the large scan-size AFM image is shown in transparent colors.

Single-particle SERS measurements are performed by correlation of AFM images and Raman maps as illustrated in Fig. 2. For SERS experiments a confocal Raman microscope and a 532 nm excitation laser is used resulting in resonant excitation of TAMRA or Cy3. AFM (Fig. 2, blue frame) and SERS images (Fig. 2, red frame) are superimposed by means of a marker on the Si surface. This approach allows for a direct assignment of SERS signals to specific DNA–AuNP hybrid structures. In this way the effect of NP structure modification on the SERS enhancement is revealed.

**Fig. 3:**
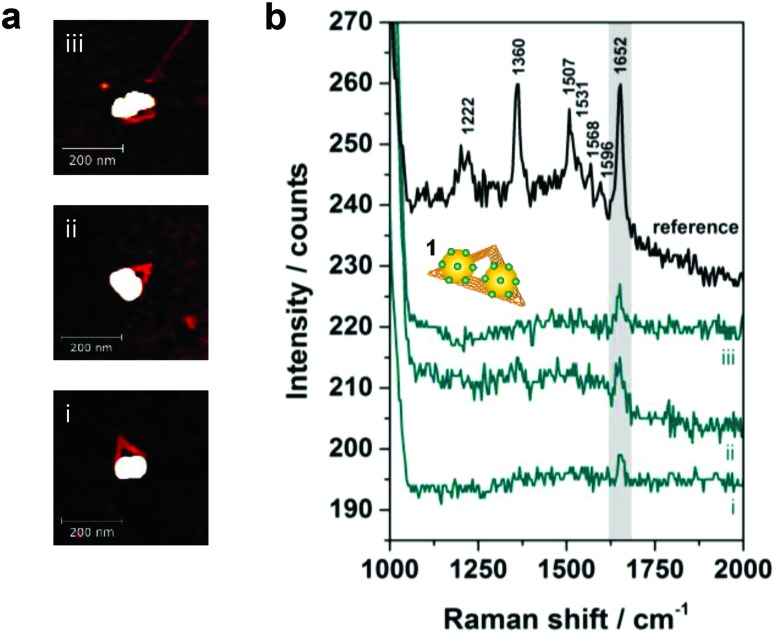
Single nanostructure correlations of 40 nm AuNP dimers covered with TAMRA-modified ssDNA (structure **1**). (a) AFM images of three individual DNA–AuNP hybrids. (b) SERS spectra of the nanostructures i–iii showing the most prominent TAMRA peak at 1652 cm^–1^ (cyan spectra). For comparison a reference spectrum containing characteristic TAMRA bands at 1222, 1360, 1507, 1531, 1568, 1596 and 1652 cm^–1^ is shown (black spectrum). The number of TAMRA molecules contributing to each of the spectra i–iii is estimated to be approximately 40. SERS experiments were performed with laser excitation at 532 nm, laser power of 900–1000 μW and 2 s integration time.

In Fig. 3 correlated AFM images and SERS spectra for single DNA origami substrates functionalized with a 40 nm AuNP dimer covered with TAMRA-modified DNA (structure **1**) are shown. The maximum gap size of approximately 14 nm between the two individual particles is estimated by considering the number of DNA bases located in the DNA origami between the two center positions of the particles (see Fig. S1 in the ESI† for details). Within the remaining structural flexibility of the AuNPs connected to the DNA origami platforms the AuNPs might approach each other slightly during the drying process due to a temporarily increased salt concentration. Thus, the minimal gap size is determined by the DNA coating which is assumed to be 2.5 nm per AuNP. In Fig. 3a AFM images of three individual and well-defined DNA–AuNP hybrids (i, ii, iii) are presented (height profiles shown in Fig. S2 (ESI†)). The corresponding SERS spectra are shown in Fig. 3b (cyan spectra). In all three cases the most prominent TAMRA band at 1652 cm^–1^ has been detected with an intensity of approximately 5–10 cts (highlighted in grey). For comparison a reference spectrum obtained from single 60 nm AuNPs covered with TAMRA-modified DNA is shown, which contains the characteristic TAMRA bands at 1222, 1360, 1507, 1531, 1568, 1596 and 1652 cm^–1^ (black spectrum). Since single 40 nm AuNPs covered with TAMRA are found to give extremely weak SERS signals (see Fig. S3 (ESI†)) under the presently applied conditions we conclude that the detected signal for structures i–iii mainly arises from TAMRA molecules located in the hot spot formed in-between the two particles. To estimate the number of TAMRA molecules that contribute to each of the three SERS spectra we assume that one 40 nm AuNP is covered with 430 oligonucleotides.^28^ Furthermore, half of the DNA coating strands are modified with a TAMRA molecule and we assume about 10% of all coating strands being located in the hot spot. Therefore, a maximum of approximately 40 TAMRA molecules contribute to each SERS spectrum (i–iii) presented in Fig. 3b. It should be emphasized that among all correlated hybrids with structure **1** only those revealing the strongest SERS signals are presented. Thus, the dimers i–iii are expected to have smaller gap sizes than 14 nm.

**Fig. 4:**
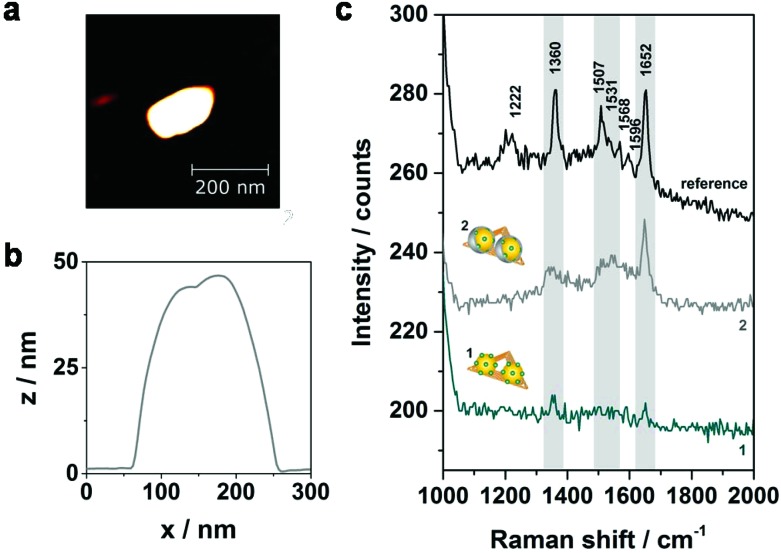
Comparison of correlated AFM images and SERS spectra before and after the silver enhancement process. (a) AFM image of one selected DNA–AuNP hybrid with silver shell (structure **2**) after 3 min of incubation (LI silver). (b) The height profile of the dimer shown in (a) indicates the growth of the silver shell. (c) Corresponding SERS spectra of the dimer shown in (a) before (cyan spectrum, 1) and after (grey spectrum, 2) electroless silver deposition. Spectrum 1 exhibits only the two most characteristic TAMRA bands at 1360 cm^–1^ and 1652 cm^–1^ (highlighted in grey). In comparison to spectrum 1 the overall SERS intensity in spectrum 2 is increased due to the silver shell. Furthermore, two additional spectral features at 1507 cm^–1^ and 1531 cm^–1^ arising from the TAMRA dyes become detectable for the core–shell system. SERS experiments were performed with laser excitation at 532 nm, laser power of 900–1000 μW and 2 s integration time.

To further improve the Raman signal enhancement an additional Ag layer is grown on the AuNPs which is expected to result in an increase of the electromagnetic field enhancement for two reasons: (a) Ag exhibits a better enhancement performance in the visible range of the electromagnetic spectrum,^29^ (b) the gap size between the two NPs is reduced upon the silver shell growth. In order to compare the effect of the silver shell, SERS and AFM data of selected nanostructures have been collected before and after the silver enhancement process (Fig. 4). In Fig. 4a an AFM image of one representative hybrid structure with silver shell is shown. The DNA origami triangle is most probably hidden beneath the Au–Ag core–shell structure in the AFM image. Since the vertical resolution in AFM images is higher than the lateral resolution by a factor of up to 100 the shell thickness can be determined from the height difference in the associated cross sections. The height profile shown in Fig. 4b indicates a thickness of the silver shell of approximately 10 nm in vertical direction (see height profiles of analogue dimers without silver shell in Fig. S2 (ESI†) for comparison). In Fig. 4c the corresponding SERS spectra are shown demonstrating an overall increase of the SERS intensity after electroless silver deposition (grey spectrum). In addition to the bands at 1360 cm^–1^ and 1652 cm^–1^ two other characteristic spectral features for TAMRA at 1507 cm^–1^ and 1531 cm^–1^ become visible (highlighted in grey). Principally, the effect of photobleaching is observed upon consecutive laser exposures under the here applied conditions. Therefore, the detected SERS signals after silver deposition are expected to result even from a smaller number of TAMRA molecules compared to the initial measurement. Although this experiment demonstrates an increase of the SERS intensity upon silver enhancement the origin of the SERS signals in structure **2** is difficult to reveal since the dye molecules are completely embedded in the silver shell. This may lead to charge transfer processes between Ag and the TAMRA molecules resulting in possible contributions from chemical enhancement. Moreover, with regard to the silver shell thickness of approximately 10 nm also DNA strands from the AuNP coating as well as from the DNA origami template might be embedded in the silver shell. Although the Raman cross section for TAMRA is considerably higher than the Raman cross sections for DNA for some hybrids clear SERS bands arising from the DNA can be detected (see Fig. S4 and Table S1 (ESI†) for an example). This observation is ascribed to two effects: on the one hand, the number of individual DNA bases incorporated in the silver shell is significantly higher in comparison to the number of TAMRA molecules. On the other hand, the two AuNPs are fused together upon silver shell growth resulting in a rod-like plasmonic particle. This can also be seen in scanning electron microscopy (SEM) images shown in Fig. S5a.† Since metal rods are known to provide the maximum electromagnetic enhancement at the particle tips^30^ new hot spots are created that provide better SERS enhancement for the DNA located at the end of the tips than for the embedded TAMRA molecules. Consequently, core–shell systems with reduced silver shell thicknesses are necessary in order to reduce the amount of embedded DNA in the shell as well as to avoid the relocation of hot spots. To improve the control of the shell size another silver enhancement kit (HQ silver, Nanoprobes) was used for subsequent experiments since it is characterized by a thickening agent to retard the deposition rate.

**Fig. 5:**
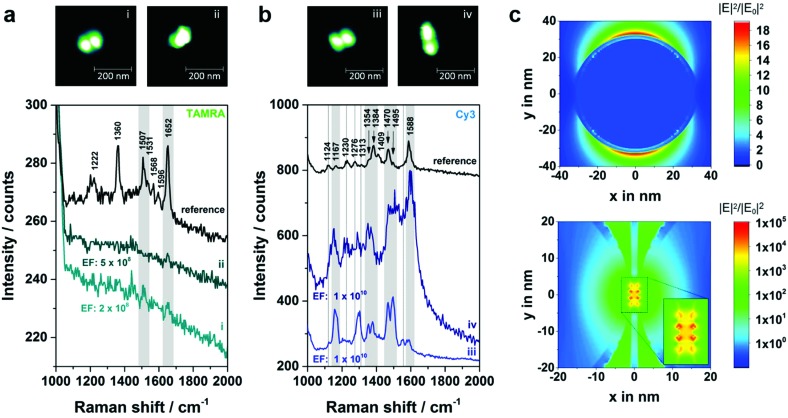
Experiments at the single-molecule level. (a) Correlated AFM and Raman study for structure **4a** with two (dimer i) or only one (dimer ii) TAMRA molecule incorporated. In both cases peaks arising from TAMRA have been detected (highlighted in grey). (b) Analogue experiment to (a) by using a single Cy3 as analyte molecule (structure **4b**). Several Cy3 bands are visible in the SERS spectra of both dimers iii and iv (highlighted in grey). Additionally, the dotted lines mark spectral positions of Cy3 which can either be detected for dimer iii or dimer iv. SERS experiments were performed with laser excitation at 532 nm, laser power of 400–500 μW and 10 s integration time. (c) FDTD calculations showing the distribution of the electromagnetic field enhancement that can be expected for a single 60 nm AuNP (top) as well as for a dimer consisting of two 60 nm AuNPs with a 2.5 nm silver shell (below). The dimer is separated by the 2 nm thick DNA origami template that is arranged concentrically in the simulation. For excitation a 532 nm laser line is assumed.

To explore the suitability of DNA origami based SERS substrates for the detection of single molecules the design was optimized taking the following aspects into account (structures **3** and **4**; strategy shown in Fig. 1b): first, placing the single dye molecule with high accuracy in the hot spot is a crucial condition since the EF decreases strongly within distances of a few nm away from the NP surface.^31^ This condition is fulfilled by attaching the two individual AuNPs to opposite sides of the DNA origami and placing the dye molecule into the axis in between the AuNPs. Moreover, in this arrangement the initial gap size is reduced to 7 nm (assuming 2 nm thickness for the DNA origami template and 2 × 2.5 nm thickness for the DNA coating surrounding the AuNPs). Second, finite-difference time-domain (FDTD) calculations revealed that Au–Ag-core–shell NP dimers (2.5 nm Ag shell and 2 nm gap size) with 60 nm Au cores show a superior electromagnetic field enhancement compared to 40 nm or 80 nm Au cores (see Fig. 5c and S7 (ESI†)). Consequently, the initial size of the AuNPs was increased to 60 nm and a thin Ag coating was added, which further reduces the gap (see Fig. S5b and S5c†). Third, an additional (-fourth) anchor point per AuNP was introduced to the DNA origami template in order to reduce the flexibility of the AuNP attachment position.

In Fig. 5a correlated Raman spectra and AFM images for structure **4a** (TAMRA as analyte molecule) are presented. Initially, an experiment with two TAMRA molecules placed into the gap has been performed (dimer i). In the corresponding AFM image the triangular DNA origami is no longer visible which is valid for all hybrids with structure **3** or **4**. This is due to the fact that the AuNP dimers are immobilized on the Si surface, while the DNA triangle can no longer lie flatly on the surface resulting in a twisted arrangement of the hybrid structures (see AFM image of a corresponding dimeric structure with two 40 nm AuNPs in Fig. S6†). The corresponding SERS spectrum for dimer i (light cyan spectrum) exhibits a weak band at 1652 cm^–1^ (highlighted in grey) which originates from a maximum of both TAMRA molecules located in the hot spot. Furthermore, the same peak was detected for only one TAMRA molecule incorporated in the DNA origami substrate (dimer ii). Additionally, a second weak band that is assigned to TAMRA is visible at 1507 cm^–1^ (also highlighted in grey). These experiments demonstrate that the detection of TAMRA at a single-molecule level is possible using the optimized hybrid structure **4a**. However, the use of TAMRA suffers from very low SERS intensities (with signal-to-noise ratios of 3.6–6.3 for the strongest TAMRA signals) and the fact that the detection is mainly based on the presence of a single peak at 1652 cm^–1^.

In order to confirm the single-molecule detection of the analyte molecule the same experiment was repeated with one Cy3 molecule incorporated into the DNA–Au–Ag hybrid structure instead of TAMRA (structure **4b**, Fig. 5b). The comparison of both single-molecule SERS spectra reveals that most spectral peak positions coincide for dimer iii and iv. All these bands at 1167, 1354, 1384, 1470, 1495 and 1588 cm^–1^ (highlighted in grey) are also visible in the reference (black spectrum) and thus can be assigned to Cy3. Some spectral features matching the reference spectrum are only present either in the SERS spectrum of dimer iii or in the one for dimer iv (marked with dashed lines). In the case of dimer iii (light blue spectrum) an additional band at 1124 cm^–1^ as well as overlapping bands in the range between 1270–1310 cm^–1^ have been detected, which arise also from the Cy3 molecule. The band at 1553 cm^–1^ is only visible as a shoulder in the reference spectrum. Another reference spectrum for Cy3, which confirms the presence of that band can be found in the ESI (Fig. S8†). In contrast, the SERS spectrum for dimer iv (dark blue spectrum) reveals a spectral feature at 1230 cm^–1^ arising from Cy3 that is only very weakly present in the spectrum of dimer iii. The observed differences between the recorded spectra (absence or presence of certain bands as well as intensity ratios) are ascribed to slight conformational variations in every individual structure. On the one hand, the position of the dye relative to the axis of the dimer is certainly different for every hybrid structure, which might lead to preferential enhancement of specific bands. On the other hand, the size ratio between Au core and Ag shell slightly differs which has been reported to have an effect on the plasmon coupling.^32^ The high background in the SERS spectrum of dimer iv (Fig. 5b, dark blue line) which is visible for some nanostructures is most likely caused by some residues from the Ag enhancement solution. In summary, the experiment shows that using Cy3 as analyte molecule gives a clear evidence of single-molecule SERS for two reasons: (a) the intensity of the SERS bands is considerably higher than for TAMRA, (b) the detection of a single Cy3 molecule is based on at least six bands (detected for both dimers iii and iv).

In order to find out whether single-molecule SERS sensitivity can be expected for the structures **4a** and **4b** EFs have been estimated, which are generally based on a comparison of the SERS signal with that of the same molecule in normal Raman conditions.^31^ Recording a normal Raman spectrum of TAMRA and Cy3 at 532 nm is hampered by a strong fluorescence background. Thus, the characterization of a reference system with known EF is necessary. Since SERS spectra of a single 60 nm AuNP covered with dye containing DNA (sequence: 5′-(TTT)_4_TX-SH-3′; X = TAMRA or Cy3) show clear bands with an acceptable signal-to-noise ratio this system was chosen to serve as a reference, whose EF was reported previously.^33^ First, the number of dye molecules per NP has been determined using a fluorescence based approach following the protocol of Hurst *et al.* ^34^ Second, the average SERS intensity for a single AuNP has been determined by correlating AFM and SERS data for approximately 15 TAMRA and 15 Cy3 labeled NPs. In the case of TAMRA the intensities of the band at 1652 cm^–1^ have been averaged whereas for Cy3 the bands at 1470 cm^–1^ and at 1588 cm^–1^ serve as references. Third, the EF for a single 60 nm AuNP was assumed to be 9 × 10^5^ according to Hong and Li.^33^ Relating the EF from the literature to the reference system and in turn to all individual hybrids shown in Fig. 5 reveals an estimated EF for every single dimer i–iv (see Fig. S9 and S10 (ESI†) for detailed information). All relevant values used for the EF estimation are summarized in Table 1. It turned out, that the EFs for the Cy3 containing systems (dimer iii and iv) are of the order of 10^10^, that is one order of magnitude higher than the EFs for the systems with TAMRA (dimer i and ii). This can be explained by a higher Raman cross section of Cy3 in comparison to TAMRA within the hot spot.

**Table 1:** Experimentally obtained data from SERS and fluorescence measurements used for the estimation of EFs

TAMRA				
System	*I* (1652 cm^–1^)/cts	Nr. TAMRA/system	*I* (1652 cm^–1^)/cts (per TAMRA)	EF
60 nm AuNP [TAMRA] (reference)	28	4805 ± 556	5.83 × 10^–3^	9 × 10^5^ ^*a*^
Dimer i	3	2	1.5	2 × 10^8^
Dimer ii	3	1	3.0	5 × 10^8^

Cy3				
System	*I* (1470 cm^–1^)/cts	Nr. Cy3/system	*I* (1470 cm^–1^)/cts (per Cy3)	EF
60 nm AuNP [Cy3] (reference)	30	3745 ± 672	8.01 × 10^–3^	9 × 10^5^ ^*a*^
Dimer iii	119	1	119	1 × 10^10^
Dimer iv	130	1	130	1 × 10^10^

System	*I* (1588 cm^–1^)/cts	Nr. Cy3/system	*I* (1588 cm^–1^)/cts (per Cy3)	EF
60 nm AuNP [Cy3] (reference)	40	3745 ± 672	1.07 × 10^–2^	9 × 10^5^ ^*a*^
Dimer iii	30	1	30	3 × 10^9^
Dimer iv	65	1	60	5 × 10^9^

^*a*^Reference EFs adopted from ref. 33.

In order to confirm the experimentally estimated EFs FDTD simulations for a single 60 nm AuNP (Fig. 5c, top) as well as for a 60 nm AuNP dimer (2.5 nm Ag shell, gap size 2 nm) (Fig. 5c, below) have been performed assuming excitation with 532 nm. In the case of the dimer the area with EFs in the range of 10^7^ or higher is expanded over approximately 5 nm in *y* direction reaching a maximal EF of 10^10^. Thus, the localization of a single dye molecule incorporated into the DNA origami structure within the hot spot is highly probable. Moreover, the experimentally estimated EFs coincide with the simulated ones. However, the EFs revealed by FDTD simulations are only based on the electromagnetic enhancement and do not consider additional effects such as the contribution due to resonant excitation of the analyte which can result in an increase of the intensity up to 5 orders of magnitude.^35^ On the other hand, the simulation is based on the excitation with light polarized along the dimer axis. Since in the experiment non-polarized light was used the simulation overestimates this contribution. Additionally, the experimentally estimated EFs are based on the EF from ref. 33 which has been determined using a 647 nm laser for excitation. Since the EF is directly correlated to the excitation wavelength the listed values only represent a first approximation. However, it should be noted that for 532 nm excitation the electromagnetic field enhancement is higher (see FDTD simulations in Fig. S11 (ESI†) for 647 nm excitation) resulting in a total EF of a single 60 nm AuNP that is about 10 times higher. Fig. S12† shows the simulated absorption and scattering spectra of the Au–Ag core–shell nanostructures, which exhibit maximum cross sections at 549 nm and 561 nm, respectively, corresponding roughly to the excitation laser used in the current experiment (532 nm).

## Experimental section

### Synthesis of DNA origami structures

Triangular DNA origami structures (design published by Rothemund^1^) were synthesized as previously described^36^ using the M13mp18 virus strand (New England Biolabs) as scaffold. For AuNP attachment either three (structures **1** and **2**) or four (structures **3a** (**4a**), **3b** (**4b**)) staple strands per AuNP were extended at the 5′-end by the capture sequence 5′-(AAA)_8_T_4_-3′. The positions of the capture strands were t-3s6e, t-3s8g, t-5s8g (according to the nomenclature used by Rothemund) and t5s6e, t5s8g, t7s8g (for structures **1** and **2**) or t-1s6e, t1s6i, t-1s8g, t1s8i and t-2s5f, t-2s7f, t2s5f, t2s7f (for structures **3a** (**4a**), **3b** (**4b**)). For incorporation of one single TAMRA molecule in the DNA origami design (structure **3a** (**4a**)) a modified capture strand t1s6i carrying a TAMRA functionality at the 3′-end was used. For the DNA origami with one Cy3 molecule (structure **3b** (**4b**)) the staple strand t-1s6i with a Cy3 modification at the 5′-end was incorporated. Except of all extended or dye-modified sequences (ordered from metabion) all ssDNA staple strands were purchased from Integrated DNA Technologies. The annealing process from 80 °C to 8 °C was realized using a Primus 25 advanced thermal cycler (Peqlab). In order to remove excess staple strands the resulting DNA origami solution (approx. 35 μL volume) was spin filtered two times at 3830 *g* for 10 min using Amicon Ultra-0.5 filters (100 kDa MWCO, Millipore). In the first run 200 μL (in the second run 300 μL) of 1× TAE with 10 mM MgCl_2_ were added to the DNA origami solution. The final concentration of the DNA origami structures was determined *via* UV-Vis absorption spectroscopy (NanoDrop 2000, Thermo Scientific) to be in the range of 25–60 nM.

### DNA-coating of AuNPs

Citrate-capped AuNPs with a size of 40 nm or 60 nm were purchased from BBI Solutions. 40 nm AuNPs were modified similar to the protocol of Ding *et al.* ^37^ whereas 60 nm AuNPs were coated following the protocol of Zhang *et al.* ^38^ with slight modifications concerning concentrations, reaction times and the use of 0.02% sodium dodecyl sulfate (SDS, Sigma Aldrich) as an additional stabilizing agent (see the ESI† for complete coating procedures). Finally, the concentration of as-prepared AuNPs was quantified by measuring the absorbance at 527 nm (for 40 nm AuNPs) or 538 nm (for 60 nm AuNPs) (NanoDrop 2000, Thermo Scientific). Related molar extinction coefficients have been calculated to be 1.03 × 10^10^ M cm^–1^ (40 nm AuNPs) or 3.96 × 10^10^ M cm^–1^ (60 nm AuNPs) using the equation suggested by Liu *et al.* ^39^

### DNA hybridization

The hybridization process between ssDNA capture strands protruding from the DNA origami template and ssDNA coating strands located at the surface of the AuNPs was realized in solution with a synthesis scale of 10 μL. Therefore, the DNA origami solution and the AuNP solution were mixed in a ratio of 1 : 1 (approx. 0.4 nM). Additionally, 1× TAE buffer was added and the final concentration of MgCl_2_ was set to 10 mM. Using the Thermocycler a temperature program was applied to the mixture keeping the solution at 45 °C for 41 min and afterwards cooling down to 25 °C in steps of 2 °C within 20 min.

### Preparation of Si substrates

Si samples (p-type, (100), CrysTec) were cut into pieces (1 × 1 cm^2^ in size), then rinsed with 4 mL ethanol (pure, Sigma Aldrich) and with 4 mL Milli-Q-water and finally dried in a stream of compressed air. Next, the Si samples were put under a UV lamp (*λ* = 254 nm; Vilber Lourmat) for 10 min to increase the hydrophilicity of the surface. A 0.4–1.0 μL drop of the DNA–AuNP solution was applied on a Si sample followed by the addition of 40 μL of 10× TAE with 100 mM MgCl_2_. During the incubation time of 60 min Si samples were stored in a box with high humidity in order to prevent the drop from drying. Finally, the substrates were rinsed with 4 mL of 1 : 1 Milli-Q-water/ethanol (pure) and dried with compressed air.

### Electroless silver deposition

For electroless silver deposition commercial enhancement kits from Nanoprobes were used (LI Silver (structure **2**) and HQ silver (structures **4a**, **4b**)). As recommended by the manufacturer the enhancement processes were performed at room temperature and under darkened light conditions. The either two (LI silver) or three (HQ silver) solutions were mixed carefully in ratios of 1 : 1 or 1 : 1 : 1 and subsequently 20 μL of the mixture were applied to the DNA–AuNP hybrids adsorbed on Si wafers. After 3 min (LI silver) or 60 s (HQ silver) of deposition time the wafers were rinsed and quickly dipped in Milli-Q-water and dried with compressed air. For single-molecule SERS measurements HQ silver was used since it is characterized by a thickening agent to retard the deposition rate and thus to increase the control over the shell size.

### Superposition of AFM and SERS images

In order to enable the superposition of recorded AFM and SERS images a small part of the Si wafer was marked with a slight scratch using a diamond knife. Subsequent AFM and SERS experiments were performed by mapping an area of 20 × 20 μm^2^ including a part of the scratch.

### AFM imaging

AFM imaging was performed using a Nanosurf FlexAFM with two compatible scan heads either for large-area imaging up to 100 μm^2^ (Fig. 2) or for higher resolution (Fig. 2–5). In both cases Tap150Al-G cantilevers (Budget Sensors) with a force constant of 5 N m^–1^ operating in tapping mode have been used. For AFM data visualization and analysis the software Gwyddion 2.34 (freeware) was used.

### Raman imaging

SERS imaging was performed with a confocal Raman microscope (WITec alpha300) equipped with an upright optical microscope. For excitation a 532 nm laser was used that was coupled into a single-mode optical fiber and focused through a 100× objective (Olympus MPlanFL N, NA = 0.9) to a diffraction-limited spot (1.3 μm^2^) on the Si sample. Due to the size of the laser spot only NP structures without any other potential SERS active NPs in the vicinity of 0.65 μm radius have been analyzed. The laser power and the integration time were set to 900–1000 μW and 2 s for dye-covered AuNPs (structures **1**, **2**) or to 400–500 μW and 10 s in the case of single-molecule measurements (structures **4a**, **4b**). The grating of the spectrograph was set to 600 g mm^–1^. For better visualization SERS spectra are vertically shifted in all diagrams presenting more than one spectrum.

### Determination of AuNPs surface coverage

The surface coverage of the AuNPs with dye-modified DNA was determined following the protocol of Hurst *et al.* ^34^ First, the concentration of AuNPs was quantified by UV-Vis absorption spectroscopy. In order to remove all DNA coating strands from the AuNP surface 10 μL of the AuNPs stock solution were incubated overnight with 10 μL of 1 M dithiothreitol (DTT, Sigma Aldrich) in 1× TAE with 10 mM MgCl_2_ [2× dilution]. The gold precipitate was removed by centrifugation at 5000*g* for 4 min. Subsequently, 17 μL of the supernatant were mixed with 493 μL of 1× TAE with 10 mM MgCl_2_ [30× dilution]. The fluorescence of the diluted supernatant containing the dye-modified DNA was measured in triplicate and compared to a calibration curve (see Fig. S10 of the ESI†). Fluorescence measurements were performed using a FluoroMax-P (Horiba Jobin Yvon). In the case of TAMRA the excitation wavelength was set to 535 nm and the emission was recorded from 550 nm to 750 nm. Cy3 was excited at 500 nm and the emission was collected from 520 nm to 700 nm. For both fluorophores an integration time of 0.2 s was used and the slit size was set to 5.0 nm.

### FDTD simulations

FDTD simulations have been performed using Lumerical FDTD Solutions 8.6.3. The thicknesses of individual layers were modelled as follows: 2.0 nm DNA origami template, 2.5 nm ssDNA coating of the AuNPs, 2.5 nm silver shell and 2.0 nm SiO_2_ substrate on a Si base. For all simulated nanostructures excitation with 532 nm was assumed. In the case of the dimeric structures the hybrids are illuminated with polarization along the axis of the dimer. All simulations are shown in equatorial plane of the particles. For the refractive indices the following values were used: 2.1 for DNA origami,^18^ 1.7 for ssDNA coating,^18^ Au and Ag (Johnson and Christy^40^), silicon and SiO_2_ (Palik^41^), 1.0 for the surrounding medium. For the calculated absorption and scattering spectra (ESI, Fig. S12†) the Ag shell was modelled using the dielectric constant according to Weast.^42^

### SEM imaging

SEM images have been recorded under high vacuum conditions with a Quanta250 (FEI) using an ETD detector in SE mode. The accelerating voltage was set to 30 kV and a spot size of 2.0 nm was used.

## Conclusion

In summary, we have demonstrated SERS from Au–Ag-core–shell NPs arranged on DNA origami substrates. A stepwise optimization strategy in terms of the AuNP size and their arrangement as well as the introduction of a silver shell has been presented in order to increase the SERS sensitivity provided by the hybrid structures. Finally, single molecules of TAMRA and Cy3 positioned in the hot spot have been successfully detected using the optimized Au–Ag-core–shell dimers. Moreover, the SERS sensitivity has been quantified by estimation of EFs for selected hybrid structures revealing values in the range between 10^9^–10^10^ for Cy3, which is one order of magnitude higher in comparison to TAMRA. The experimentally estimated EFs were found to be in good agreement with theoretical values provided by FDTD simulations.

The novel SERS substrates presented here are highly promising for biosensing applications. For instance, single proteins or DNA strands can be placed in the hot spot with high local precision in order to investigate *e.g.* protein folding^43^ or DNA strand break events. Furthermore, the plasmon resonance of the Au–Ag-core–shell NPs can be easily tuned by adjusting the size ratio between core and shell^32^ which enables the creation of tailored nanostructures for individual applications. Extending the dimeric structures by an additional NP might result in even higher EFs, especially in the case of so called nanolenses consisting of three differently sized AuNPs with specific size ratios.^44,45^ Consequently, single-molecules with much lower Raman cross-sections might become detectable using DNA–NP hybrid structures in the future.
