# Supplementary material for: DNA origami based Au–Ag-core–shell nanoparticle dimers with single-molecule SERS sensitivity
Source: Nanoscale. 2016 Feb 19;8(10):5612–20. doi: 10.1039/c5nr08674d (PMC4778414; doi:10.1039/c5nr08674d)
Supplement: Supplementary file 1 [file NR-008-C5NR08674D-s001.pdf]

Electronic Supplementary Material (ESI) for Nanoscale.
This journal is © The Royal Society of Chemistry 2016

Electronic Supplementary Information (ESI)

DNA origami based Au-Ag-core-shell nanoparticle dimers with single-molecule SERS sensitivity

Julia Prinz[1], *Christian Heck*[1,2,3], *Lisa Ellerik*[1], *Virginia Merk*[3], *Ilko Bald*[1,2,*]

[1]Institute of Chemistry, University of Potsdam, Karl-Liebknecht-Str. 24-25, 14469 Potsdam, Germany.

[2]BAM Federal Institute for Materials Research and Testing, Richard-Willstätter Str. 11, 12489 Berlin, Germany.

[3]Department of Chemistry + SALSA, Humboldt-Universität zu Berlin, Brook-Taylor-Str. 2, 12489 Berlin, Germany.

*bald@uni-potsdam.de

1. Coating procedure of AuNPs

Salt aging method

First, 5 mg of Bis(p-sulfonatophenyl)phenylphosphine dehydrate dipotassium salt (BSPP, Sigma Aldrich) were added to 15 mL of 40 nm citrate-stabilized AuNPs, covered with aluminium foil and stirred for 24 h. Next, NaCl was added to the solution until a change of color from red to purple was visible. The solution was centrifuged at 500 g for 35 min, the supernatant was discarded and 0.3 mL of 2.5 mM BSPP solution were added. The re-suspension of the AuNPs was indicated by a change of color back to red. Subsequently, 0.5 mL methanol (Sigma Aldrich) were added resulting in a third change of color to black. The solution was centrifuged at 500 g for 35 min, the supernatant was removed and AuNPs were re-suspended in 0.2 mL 2.5 mM BSPP solution. For the DNA coating process a 64000-fold excess of disulfide-modified DNA strands over AuNPs was used 13 µL of phosphinated AuNPs were mixed with 11.5 µL TAMRA-containing ssDNA (5'-$(TTT)_4$TX-SH-3' ; X = TAMRA; 100 µM) and 11.5 µL of the analogue ssDNA strand without TAMRA (5'-$(TTT)_4$T-SH-3'; 100 µM). Furthermore, 2.9 µL of 10x TAE was added and the mixture was allowed to stir overnight. The following salt aging process was realized over a long period of 7 days in order to prevent the AuNPs from precipitation. During that time volumes between 0.5 – 2.0 µL of a 2M NaCl solution were stepwise added to the AuNP solution resulting in a final NaCl concentration of 360 mM. After each step, the mixture was sonicated for 20 s and stirred until the next salt addition. Excess DNA strands were removed by spin filtering (58.4 µL of AuNP solution + 200 µL of 1x TAE with 10 mM $MgCl_2$) using Amicon Ultra-0.5 filters (100 kDa MWCO, Millipore) at 3830 *g* for 10 min. The following washing process with 200 µL of 1x TAE with 10 mM $MgCl_2$ at 3830 *g* for 10 min was performed 4 times. After the last washing step no signals arising from unbound DNA strands were detected via UV-Vis spectroscopy anymore.

pH method

First, the stock solution of 60 nm AuNPs was concentrated by centrifuging 0.4 mL of citrate-stabilized AuNPs at 1000 *g* for 10 min. The supernatant was gently removed and 4 µL of 0.2 % SDS were added to 20 µL of the concentrated AuNPs (1-2 nM). Next, 1.8 µL of the ssDNA coating strands (sequence 5'-$(TTT)_4$T-SH-3'; 100 µM) was added. The mixture was covered with aluminium foil and stirred for at least 30 min. Then, 8 µL of 50 mM citrate buffer (pH 3) were added in steps of 1-2 µL over a period of 2 h. After addition of 6.4 µL of Milli-Q-water the mixture was stirred for at least 60 min. A total volume of 5 µL NaCl solution (2.5 M) was added in steps of 1 µL within 2 h and afterwards the mixture was allowed to stir overnight. The AuNPs solution was centrifuged at 1500 *g* for 7 min and the supernatant was discarded. In order to remove unbound coating strands the mixture was washed 5 times by adding

200 µL of 1x TAE with 0.02 % SDS and 10 mM MgCl2, spinning at 1500 *g* for 7 min and discarding the supernatant.

2. Designs of the DNA origami triangles

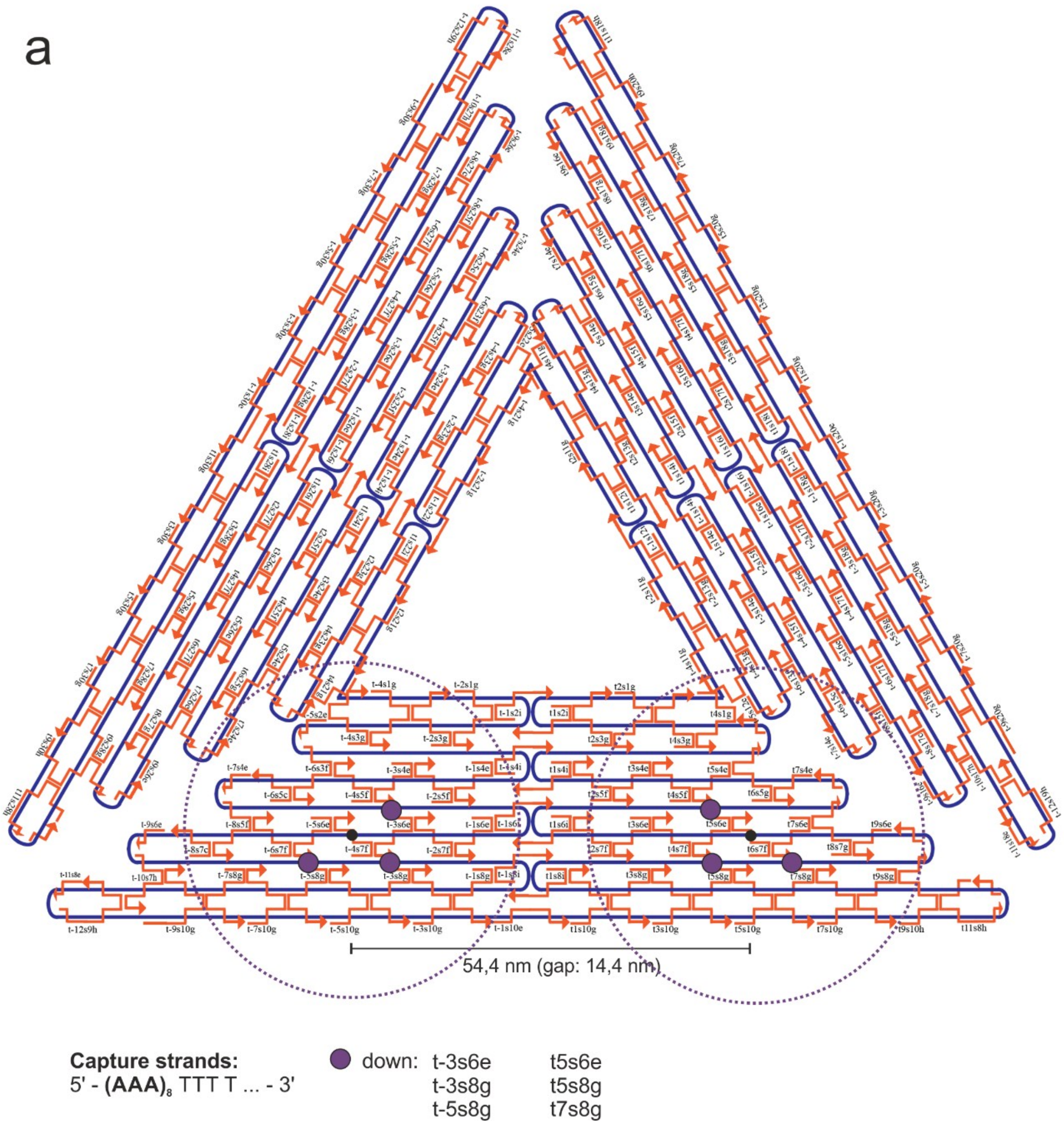

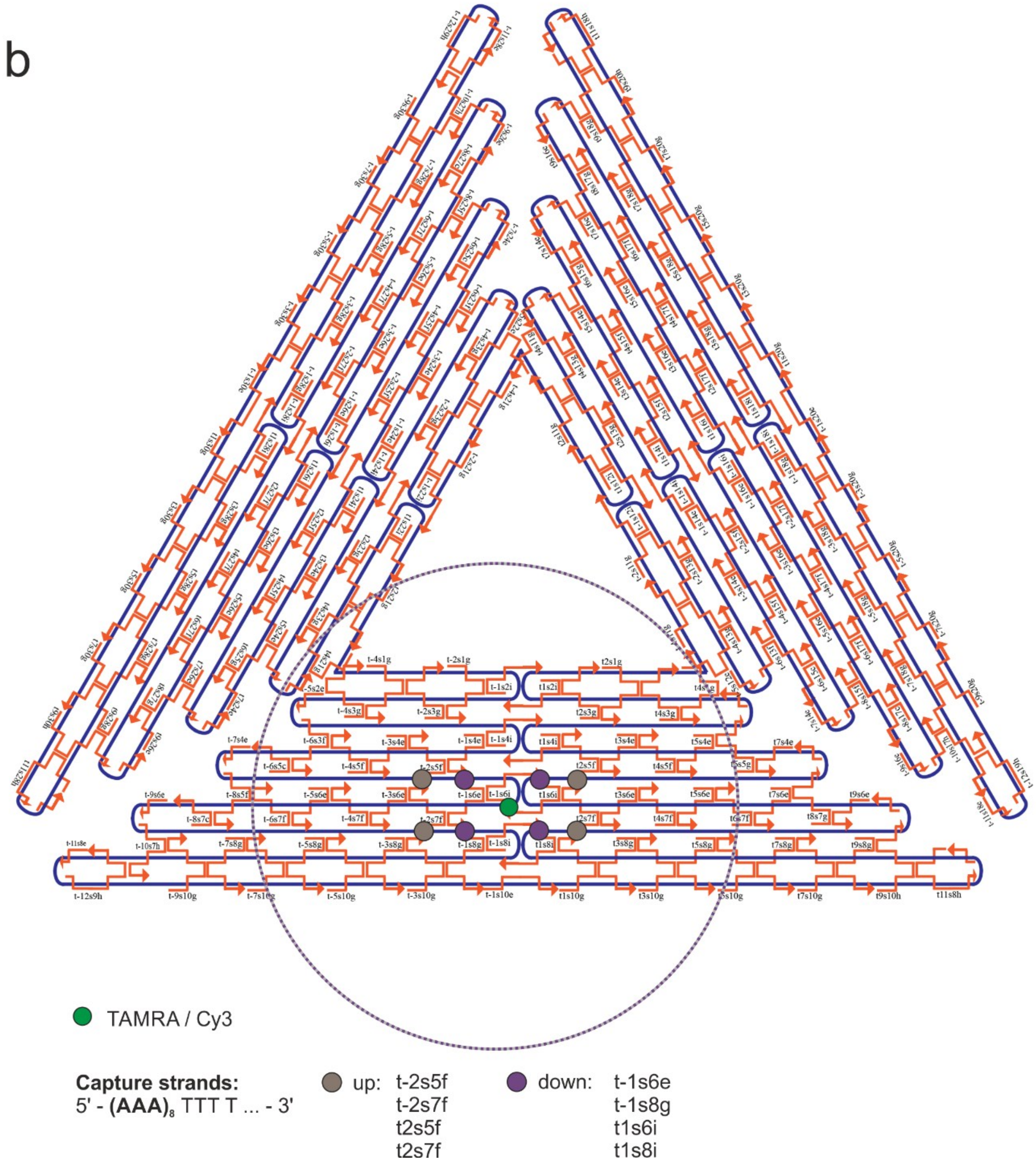

Figure S1. *Designs of DNA origami triangles* (according to the nomenclature used by Rothemund[1]. *The first design (a) was used for the attachment of two 40 nm AuNPs to one side of the DNA origami template (strategy a in Figure 1), the second design (b) for the attachment of two 60 nm AuNPs to different sides (strategy b Figure 1). The positions of capture strands are marked in grey and violet; the position for the dye molecule is marked in green.*

3. Height profiles for dimers

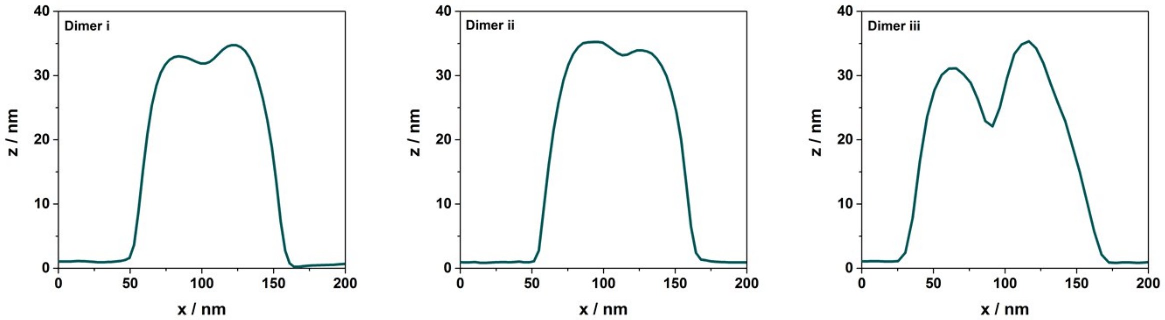

Figure S2. *Height profiles for dimers i,ii and iii shown in Figure 3 (structure **1**).*

4. SERS spectrum for single 40 nm AuNPs covered with TAMRA-modified DNA

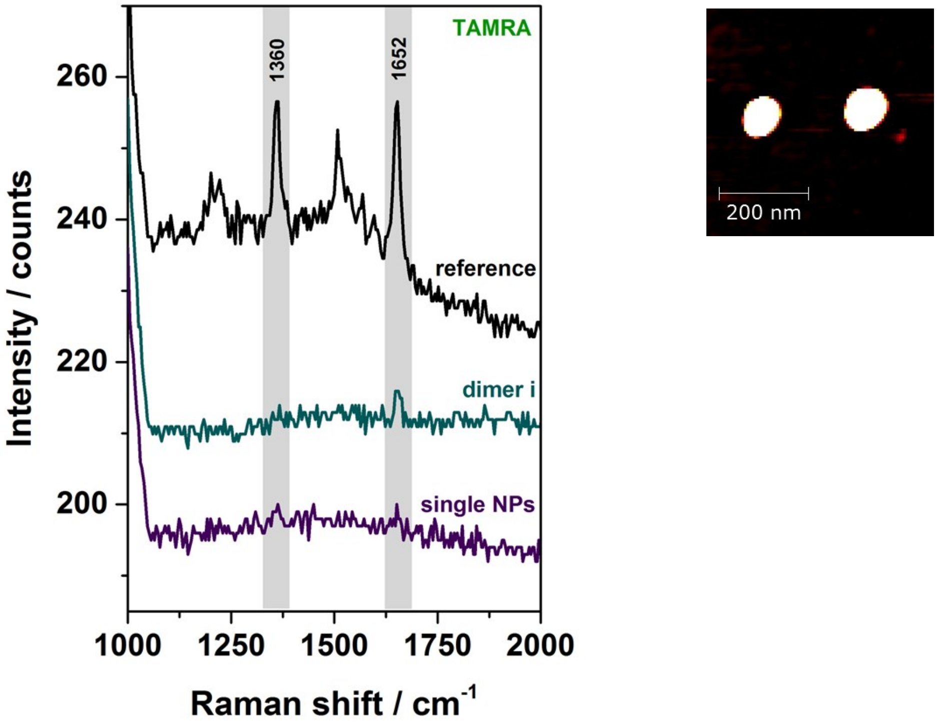

Figure S3. *Comparison of SERS spectra for a dimeric structure (structure **1**, dimer i) and two single 40 nm AuNPs (corresponding AFM image shown on the right) covered with TAMRA-modified DNA. Single AuNPs only give negligibly weak SERS signals at 1360 and 1652 cm*$^{-1}$.

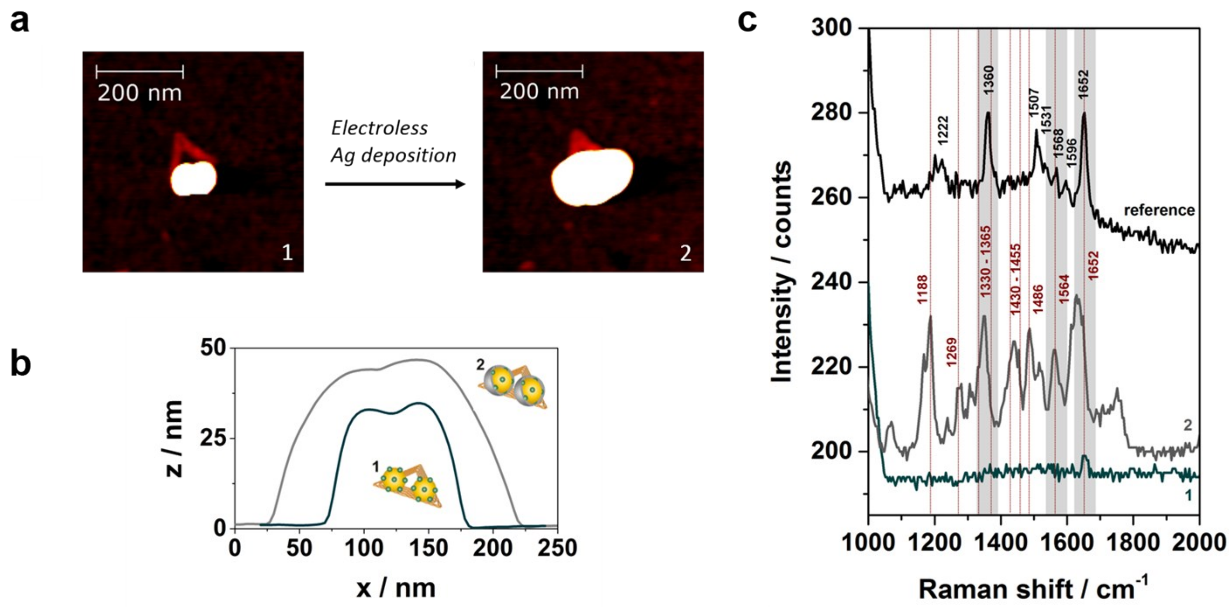

5. Assignment of additional DNA bands

Figure S4. *Example of a hybrid structure revealing DNA bands in the SERS spectrum upon electroless silver deposition. (a) AFM images of the selected DNA-AuNP hybrid without (structure **1**) and with (structure **2**) silver shell after 3 min of incubation (LI silver). (b) The height profiles of the two dimers shown in a) indicate the growth of the silver shell. (c) Corresponding SERS spectra of the dimers shown in a) before (cyan spectrum, **1**) and after (grey spectrum, **2**) electroless silver deposition. Spectral positions of TAMRA are highlighted in grey whereas bands arising from DNA are indicated by red dotted lines. In the corresponding Table S1 the assignment of all bands to certain DNA bases is summarized.*

Table S1. *Assigment of DNA bands visible in the SERS spectrum of Figure S4 (red dotted lines).*

**Raman shift / cm^{-1}**	**DNA base**	
1188	T[2]	T = thymine
1269	T, A[2]	A = adenine
1330 – 1365	T, A, G, C[2]	G = guanine
1430 – 1455	T, A, G[2]	C = cytosine
1486	A[3]	
1564	T[2]	
1652	T[2]	

6. SEM images

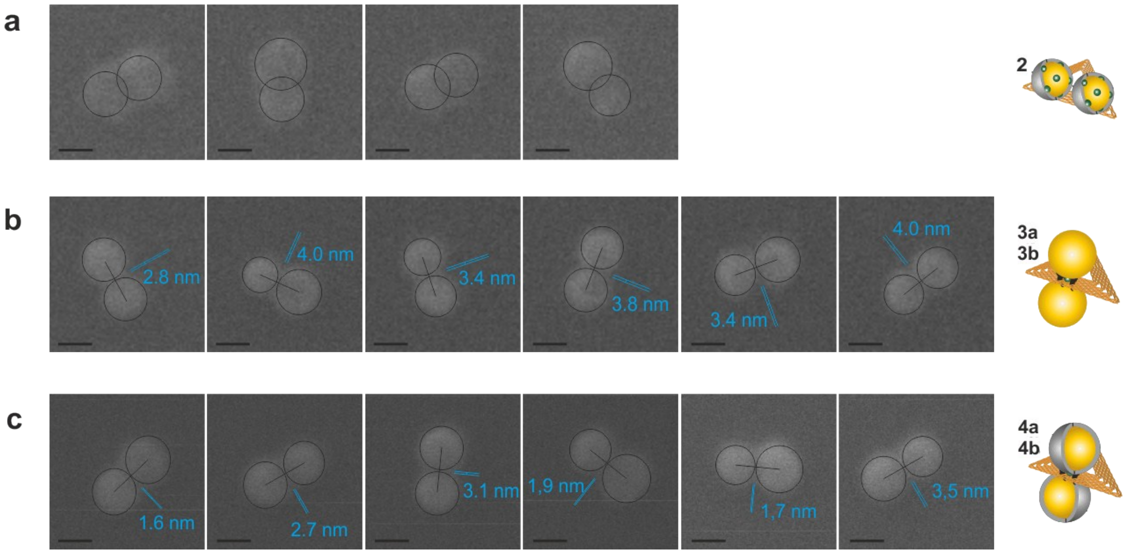

Figure S5. *Scanning electron microscopy (SEM) images of representative DNA-origami-AuNP-hybrids with different structures. (a) SEM images of hybrids functionalized with two 40 nm AuNPs on one side of the DNA origami (structure **2**) confirm that the two individual AuNPs are fused together upon electroless silver deposition resulting in gapless structures. (b,c) For hybrids of structure **3a**(**3b**) an average gap size of 3.6 nm has been determined. Due to the silver enhancement process the analogue Au-Ag-core-shell-hybrids (**4a**(**4b**)) reveal a reduced average gap size of 2.4 nm. Scale bars = 50 nm.*

7. AFM images of DNA origami substrates with two 40 nm AuNPs on different sides

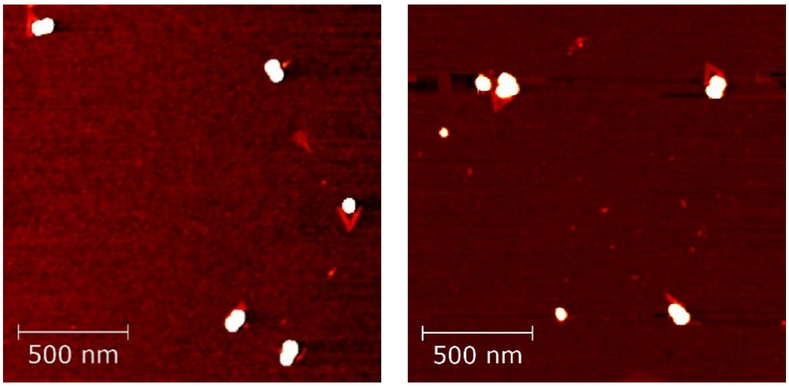

Figure S6. *AFM images of DNA origami triangles functionalized with two 40 nm AuNPs on different sides before (left) and after the silver enhancement process (right). In both cases the DNA origami substrates are clearly visible which is not the case for the analogue structures functionalized with two 60 nm AuNPs (see Figure 5 for comparison).*

8. FDTD simulations

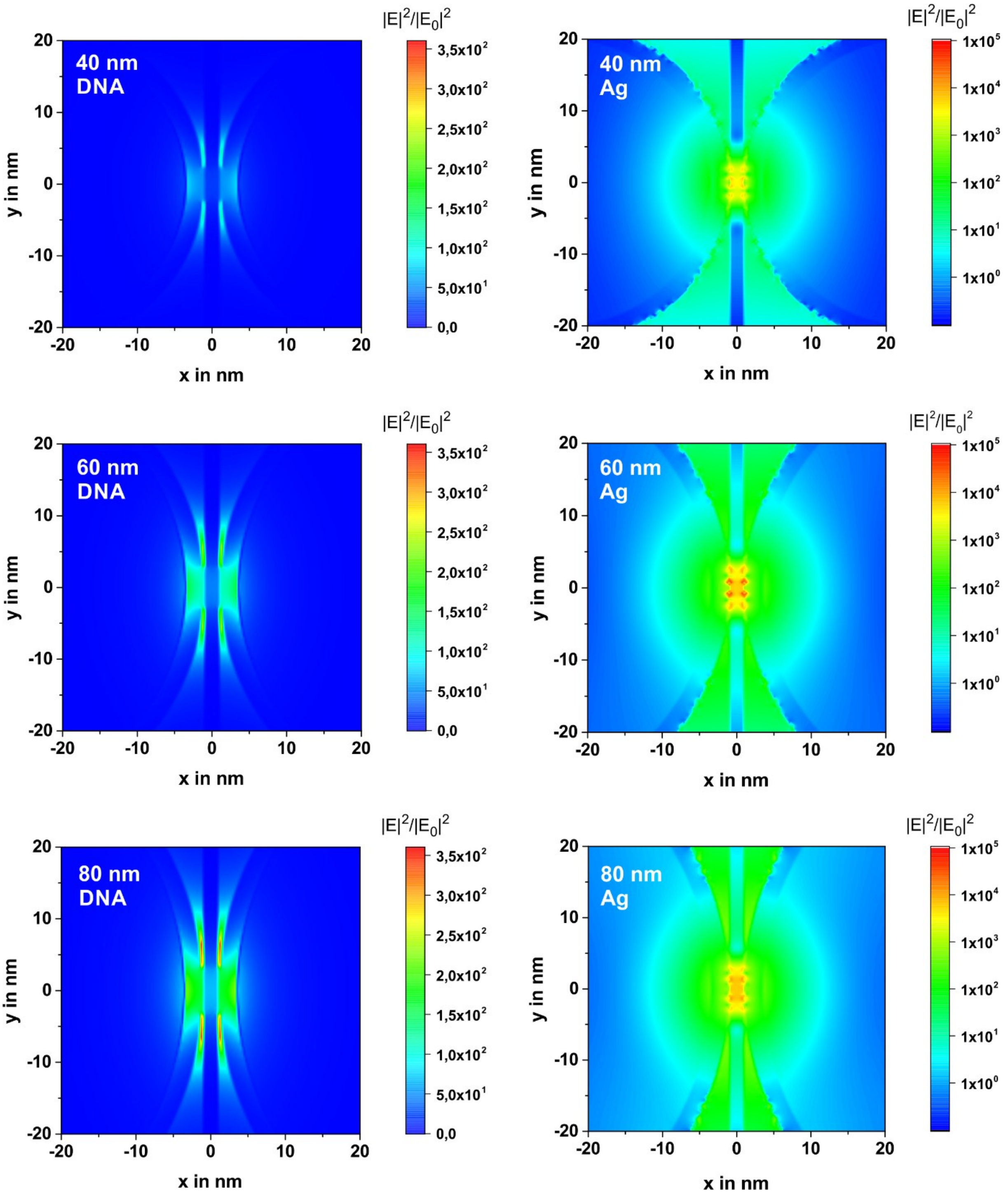

Figure S7. *FDTD simulations for 40 nm (first row), 60 nm (second row) and 80 nm AuNP dimers (third row) attached to different sides of the DNA origami template (Figure 1b), structures **3**, **4**). All dimers are separated by the DNA origami template (2.0 nm thickness) which is arranged concentrically in the simulations. For the simulations a 2.5 nm DNA coating (bare Au cores; left column) or a 2.5 nm Ag shell*

(Au-Ag-core-shell NPs; right column) is assumed. For all individual Au core sizes a significant increase of the electromagnetic field enhancement is observed upon electroless silver deposition. Comparing all simulations reveals that highest electromagnetic field enhancements can be expected for 60 nm AuNP dimers covered with a silver shell (second row, right column).

9. Reference spectra for Cy3

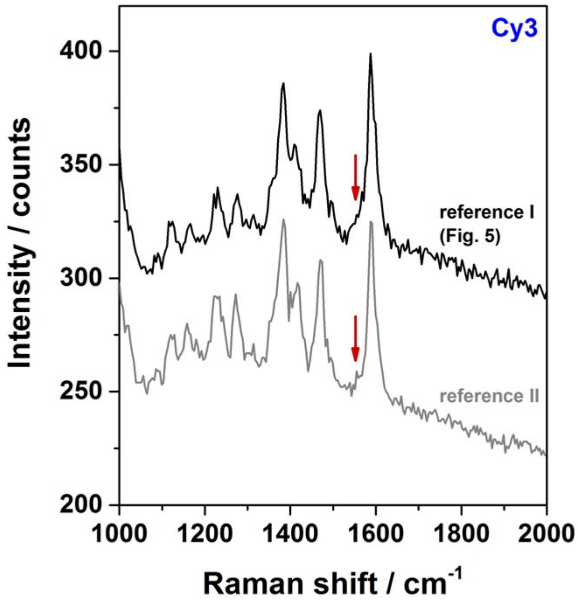

Figure S8. *Two different SERS spectra serving as a reference for Cy3. The peak at 1553 cm^{-1} (red arrow) which is only visible as a shoulder for reference I (black spectrum) is clearly detected for reference II (grey spectrum).*

10. Data for EF estimation

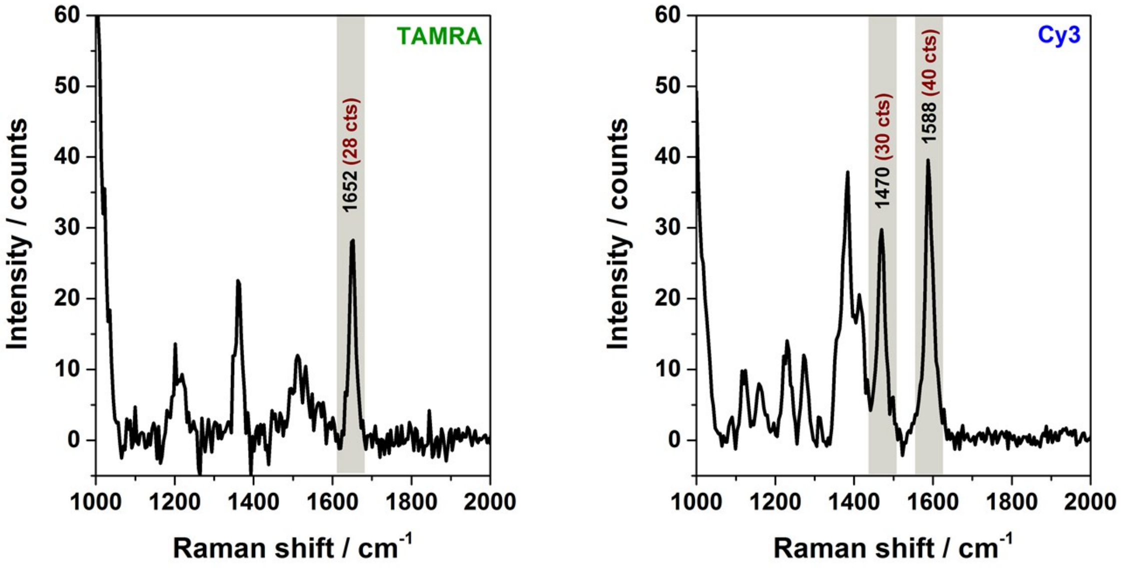

Figure S9. *Background corrected average SERS spectra of approximately 15 single 60 nm AuNPs covered with dye-modified ssDNA (5'-(TTT)$_4$TX-SH-3'; X = TAMRA (left), X = Cy3 (right)). SERS experiments were perfoFrmed using the same parameters as for the dimers in Figure 5. The SERS intensity of the highlighted bands were used for the experimental estimation of EFs (see Table 1).*

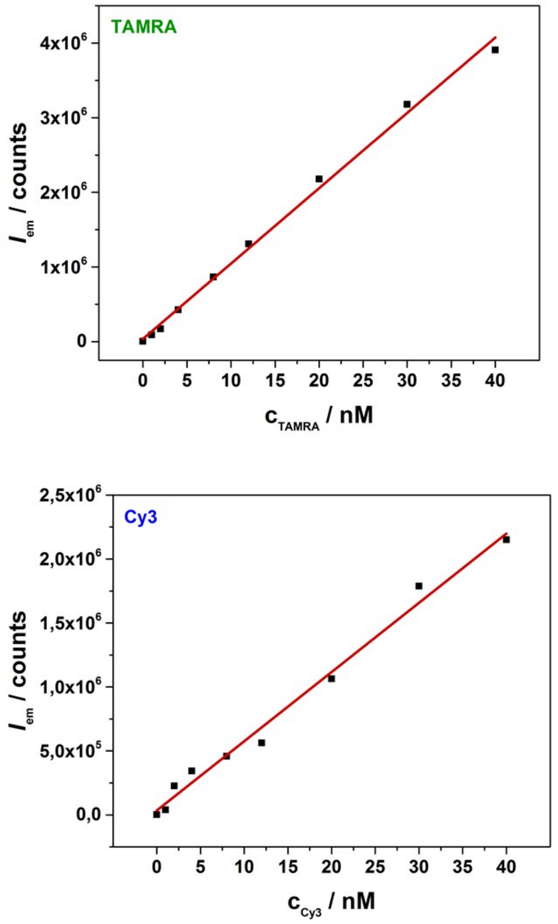

Figure S10. *Calibration curves to determine the number of dye-modified DNA strands (5'-(TTT)$_4$TX-SH-3') per 60 nm AuNP for X = TAMRA (left) and X = Cy3 (right) in order to estimate EFs for selected structures (see Table 1). The method was adopted from Hurst et al.*[4]*.*

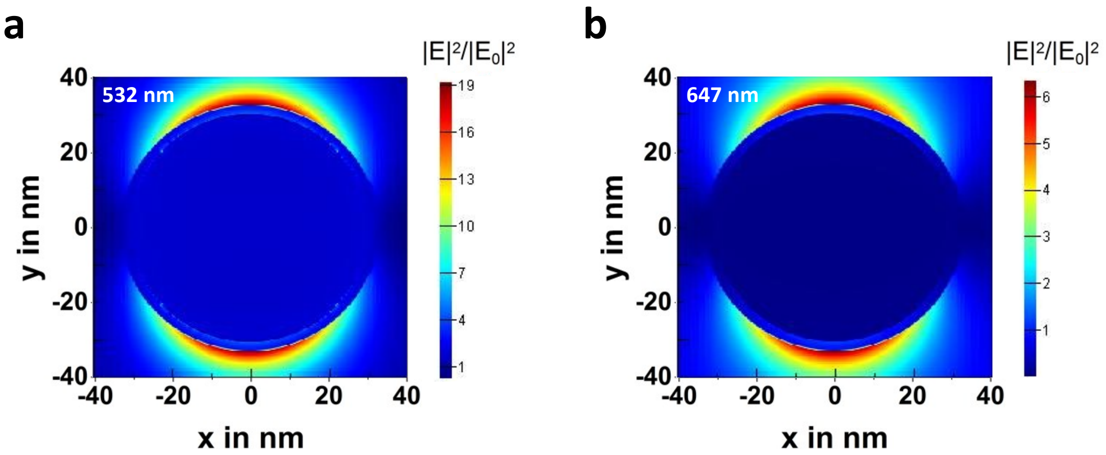

11. Comparison of FDTD simulations for different excitation wavelengths

Figure S11. *Comparison of FDTD calculations for a single 60 nm AuNP using a 532 nm (a) or a 647 nm (b) laser for excitation. The maximum electromagnetic field that can be expected is approximately 10 times higher for excitation with 532 nm.*

12. Calculated absorption and scattering spectra

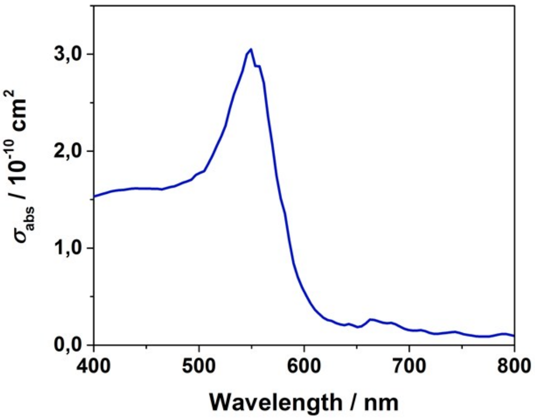

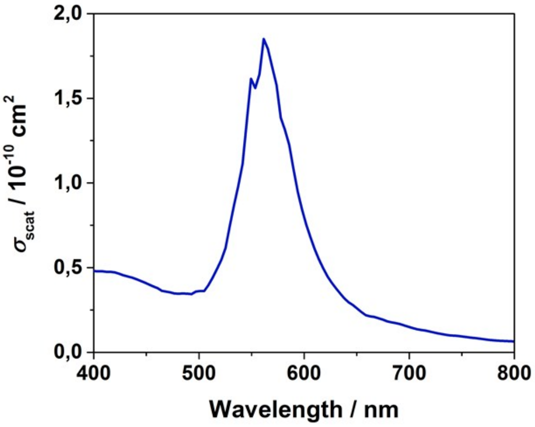

Figure S12. *Calculated absorption (left) and scattering (right) spectra for dimeric Au-Ag-core-shell-structures (60 nm Au core, 2.5 nm silver shell) separated by a gap of 2.0 nm. Calculations reveal a maximum absorption cross-section at 549 nm and a maximum scattering cross-section at 561 nm.*

References

(1) Rothemund, Paul W. K. Folding DNA to create nanoscale shapes and patterns. *Nature* [Online] **2006**, *440*, 297–302. http://dx.doi.org/10.1038/nature04586.

(2) Otto, C.; van den Tweel, T. J. J.; de Mul, F. F. M.; Greve, J. Surface-enhanced Raman spectroscopy of DNA bases. *J. Raman Spectrosc.* [Online] **1986**, *17* (3), 289–298. http://dx.doi.org/10.1002/jrs.1250170311.

(3) Thacker, V. V.; Herrmann, L. O.; Sigle, D. O.; Zhang, T.; Liedl, T.; Baumberg, J. J.; Keyser, U. F. DNA origami based assembly of gold nanoparticle dimers for surface-enhanced Raman scattering. *Nat Commun* [Online] **2014**, *5*. http://dx.doi.org/10.1038/ncomms4448.

(4) Hurst, S. J.; Lytton-Jean, Abigail K. R.; Mirkin, C. A. Maximizing DNA Loading on a Range of Gold Nanoparticle Sizes. *Anal. Chem.* [Online] **2006**, *78* (24), 8313–8318. http://dx.doi.org/10.1021/ac0613582.
